# Chronic Obstructive Pulmonary Disease and Its Acute Exacerbation before Colon Adenocarcinoma Treatment Are Associated with Higher Mortality: A Propensity Score-Matched, Nationwide, Population-Based Cohort Study

**DOI:** 10.3390/cancers13184728

**Published:** 2021-09-21

**Authors:** Yen-Chang Chen, Ming-Chang Li, Ying-Hui Yu, Chih-Ming Lin, Szu-Yuan Wu

**Affiliations:** 1 Division of Chest Medicine, Department of Internal Medicine, Lo-Hsu Medical Foundation, Lotung Poh-Ai Hospital, Yilan 256, Taiwan; 783006@mail.pohai.org.tw; 2 Department of Colorectal Surgery, Lo-Hsu Medical Foundation, Lotung Poh-Ai Hospital, Yilan 256, Taiwan; C059019@mail.pohai.org.tw (M.-C.L.); c843024@mail.pohai.org.tw (Y.-H.Y.); 3 Division of Thoracic Surgery, Department of Surgery, Lo-Hsu Medical Foundation, Lotung Poh-Ai Hospital, Yilan 256, Taiwan; 4 Department of Food Nutrition and Health Biotechnology, College of Medical and Health Science, Asia University, Taichung 413, Taiwan; 5 Big Data Center, Lo-Hsu Medical Foundation, Lotung Poh-Ai Hospital, Yilan 256, Taiwan; 6 Division of Radiation Oncology, Lo-Hsu Medical Foundation, Lotung Poh-Ai Hospital, Yilan 256, Taiwan; 7 Department of Healthcare Administration, College of Medical and Health Science, Asia University, Taichung 413, Taiwan; 8 Cancer Center, Lo-Hsu Medical Foundation, Lotung Poh-Ai Hospital, Yilan 256, Taiwan; 9 Graduate Institute of Business Administration, Fu Jen Catholic University, Taipei 242062, Taiwan; 10 Centers for Regional Anesthesia and Pain Medicine, Taipei Municipal Wan Fang Hospital, Taipei Medical University, Taipei 110, Taiwan

**Keywords:** colon, adenocarcinoma, COPD, AECOPD, all-cause mortality

## Abstract

**Simple Summary:**

This is the first study to reveal that hospitalization frequency for acute exacerbation of chronic obstructive pulmonary disease (AECOPD) before colon adenocarcinoma treatment is a severity-dependent and independent prognostic factor for overall survival in patients with stage I–III colon cancer receiving surgical resection and standard treatments. In patients with colon adenocarcinoma undergoing curative resection, those with chronic obstructive pulmonary disease (COPD) had poorer survival outcomes than had those without COPD. Hospitalization for AECOPD at least once within 1 year before colon adenocarcinoma diagnosis is an independent risk factor for poor overall survival in these patients, and a higher number of hospitalizations for AECOPD within 1 year before diagnosis was associated with poorer survival. Our study may be applied to accentuate the importance of COPD management, particularly the identification of frequent exacerbators and the prevention of AECOPD, before standard colon adenocarcinoma treatments are initiated.

**Abstract:**

Purpose: To investigate whether chronic obstructive pulmonary disease (COPD) and COPD severity (acute exacerbation of COPD (AECOPD)) affect the survival outcomes of patients with colon adenocarcinoma receiving standard treatments. Methods: From the Taiwan Cancer Registry Database, we recruited patients with clinical stage I–III colon adenocarcinoma who had received surgery. The Cox proportional hazards model was used to analyze all-cause mortality. We categorized the patients into COPD and non-COPD (Group 1 and 2) groups through propensity score matching. Results: In total, 1512 patients were eligible for further comparative analysis between non-COPD (1008 patients) and COPD (504 patients) cohorts. In the multivariate Cox regression analysis, the adjusted hazard ratio (aHR; 95% confidence interval (CI)) for all-cause mortality for Group 1 compared with Group 2 was 1.17 (1.03, 1.29). In patients with colon adenocarcinoma undergoing curative resection, the aHRs (95% CIs) for all-cause mortality in patients with hospitalization frequencies of ≥1 and ≥2 times for AECOPD within 1 year before adenocarcinoma diagnosis were 1.08 (1.03, 1.51) and 1.55 (1.15, 2.09), respectively, compared with those without AECOPD. Conclusion: In patients with colon adenocarcinoma undergoing curative resection, COPD was associated with worse survival outcomes. Being hospitalized at least once for AECOPD within 1 year before colon adenocarcinoma diagnosis was an independent risk factor for poor overall survival in these patients, and a higher number of hospitalizations for AECOPD within 1 year before diagnosis was associated with poorer survival. Our study highlights the importance of COPD management, particularly the identification of frequent exacerbators and the prevention of AECOPD before standard colon adenocarcinoma treatments are applied.

## 1. Introduction

Chronic obstructive pulmonary disease (COPD) was the third leading cause of death and seventh leading cause of disability-adjusted life years worldwide in 2019 [1,2]. However, the COPD burden becomes greater if the relationship of COPD with cancers is considered. COPD is a well-known independent risk factor for lung cancer [3,4,5]. Moreover, recent studies have suggested that regardless of the smoking status, COPD is a risk factor for several extrapulmonary solid organ cancers, including colorectal cancer [6,7]. The treatment outcome of colon cancer may be negatively affected by COPD because it increases the risk of postoperative complications, precludes patients from receiving adjuvant chemotherapy, and reduces the effectiveness of chemotherapy [8,9,10,11]. Nevertheless, to our knowledge, for patients with colon cancer who received surgical resection and standard treatments, no study has been conducted to estimate the long-term mortality risk posed by their pre-existing COPD and their COPD severity (defined as the frequency of acute exacerbation of COPD (AECOPD) associated with hospitalization within 1 year before surgical resection in this study).

In this study, we focused on colon cancer but not colorectal cancer. Colon cancer and rectal cancer are different in terms of patient characteristics, the prevalence and patterns of genetic mutations, plasma protein expression, types of hereditary cancer, blood supply and drainage, innervations, and the invasive growth of primary tumors; hence, their surgical approaches, chemosensitivity, response to and feasibility of radiotherapy, and treatment outcomes also vary [12,13,14].

This study investigated whether COPD and COPD severity affect the survival of patients with colon cancer receiving standard treatments. If COPD severity before colon cancer treatment was identified as a crucial prognostic factor for survival, determining COPD severity before colon cancer treatment and preventing COPD from progressing to AECOPD could improve survival after colon cancer treatment.

## 2. Patients and Methods

### 2.1. Study Population

In this study, cohort data were retrieved from the Taiwan Cancer Registry Database (TCRD), a nationwide population-based registry containing data of patients newly diagnosed as having cancer. The patients enrolled in this study were diagnosed as having stage I–III colon adenocarcinoma (according to the Cancer Staging Manual of the American Joint Committee on Cancer (AJCC), eighth edition [15]) between 1 January 2009, and 31 December 2018. The index date was the date on which each patient underwent surgical resection. The follow-up duration was the period from the index date to 31 December 2019. With financial support of Taiwan’s Ministry of Health and Welfare, the TCRD maintains detailed cancer-related information on patients, including disease stage, cigarette smoking habits (but not smoking intensity), treatment modalities, pathologic data, and chemotherapy regimens used [16,17]. The study protocols were reviewed and approved by the Institutional Review Board of Tzu-Chi Medical Foundation (IRB109-015-B).

### 2.2. Inclusion and Exclusion Criteria

The pathological data of patients were reviewed to confirm their diagnoses, and that no patient enrolled had distant metastases or any other cancer except colon adenocarcinoma. Those who were newly diagnosed as having colon adenocarcinoma and who underwent surgical resection were included if they were ≥20 years old and had pathologic cancer stage of I–III according to the eighth edition of the AJCC Cancer Staging Manual. Patients with high-risk pathologic features—that is, tumor invasion or adherence to adjacent organs or tissues, such as vascular, lymphatic, or perineural invasion—were included. Patients were excluded from the study if they had a history of cancer before their colon adenocarcinoma diagnosis, unknown pathologic types, missing sex data, unclear staging, and a histological type other than adenocarcinoma.

The comorbidity incidence was scored using the Charlson comorbidity index (CCI) [18,19,20]. Comorbidities were coded according to the International Classification of Diseases, 10th Revision, Clinical Modification codes [19]. Comorbidities were included in the CCI score if they were reported and assigned at the first admission or led to more than two outpatient department visits. Only comorbidities observed within 6 months before the index date were considered. Diabetes, hyperlipidemia, hypertension, COPD, end-stage renal disease (ESRD), and coronary heart disease were excluded from the CCI scores to avoid multiple adjustment in the multivariate analysis.

### 2.3. Propensity-Score Matching and Covariates

We implemented propensity-score matching (PSM) to control confounding effects before comparing all-cause mortality between the COPD and non-COPD cohorts. The cohorts were matched at a ratio of 1:2 in terms of variables such as sex, age, CCI score, diabetes, hyperlipidemia, hypertension, ESRD, coronary heart disease, cigarette smoking habit, histological differentiation grade, urbanization, obesity, chemotherapy (including all neoadjuvant and adjuvant chemotherapies), perineural invasion, lymphovascular invasion, and AJCC pathologic stages. A caliper width of 0.2 was applied for matching [21]. The robust sandwich estimator that considers clustering within matched sets was used for Cox regression. Hazard ratios were calculated through multivariate Cox regression analyses to determine whether any of the following factors were independent predictors of all-cause mortality: COPD status, AECOPD hospitalization frequency within 1 year before surgical resection, sex, age, CCI score, diabetes, hyperlipidemia, hypertension, ESRD, coronary heart disease, cigarette smoking habit, histological differentiation grade, urbanization level, obesity, chemotherapy (including all neoadjuvant and adjuvant treatments), perineural invasion, lymphovascular invasion, and AJCC pathologic stages. These potential predictors were controlled through PSM before further statistical analyses were conducted (Table 1). The primary endpoint for both COPD and non-COPD groups was all-cause mortality. Colon cancer death estimations according to the Cause of Death database are presented in Table 1.

After the inclusion and exclusion criteria were applied and PSM was conducted, 1512 patients diagnosed as having AJCC clinical stage I–III colon adenocarcinoma and who received surgical resection were eligible for further analysis. They were categorized into two groups based on their COPD status to compare all-cause mortality: one group comprised patients diagnosed as having COPD at least 1 year before undergoing surgical resection for their newly diagnosed colon adenocarcinoma, and the other group comprised non-COPD patients (defined as those who had never been diagnosed as having COPD before or throughout the study period). In addition to the comparison of all-cause mortality between COPD and non-COPD groups, we estimated the survival outcomes of patients with COPD who had different frequencies of severe acute exacerbation that resulted in hospitalization. The definition of AECOPD was that patients used the main diagnosis code of AECOPD for hospitalization and had COPD medications, including inhaled short-acting bronchodilator therapy or systemic glucocorticoids, during hospitalization for AECOPD.

### 2.4. Statistics

Comparative analyses of all-cause mortality between COPD and non-COPD groups were performed after confounders were adjusted. A *p* value of <0.05 was considered statistically significant in a two-tailed test. All survival curves were estimated using the Kaplan–Meier method, and a comparison of survival curves, stratified according to matched sets, among non-COPD, COPD, and hospitalization for AECOPD groups was performed using a stratified log rank test [22]. All statistical analyses were performed using SAS version 9.3 software (SAS Institute, Cary, NC, USA).

## 3. Results

### 3.1. PSM and Study Cohort

There were 3548 patients initially identified for further matching with non-COPD (3044 patients) and COPD (504 patients) cohorts; 57.38% of initial cohort were then excluded. Finally, 1512 patients were eligible for further comparative analysis between non-COPD (1008 patients) and COPD (504 patients) cohorts. The age distribution between the two groups was balanced (Table 1). After head-to-head PSM, the variables of the two cohorts, including sex, diabetes, hyperlipidemia, hypertension, ESRD, cigarette smoking, coronary heart disease, obesity, CCI score, histological differentiation, urbanization, perineural invasion, chemotherapy, lymphovascular invasion, and AJCC pathologic stages, were well matched and showed no statistically significant differences. Death, follow-up time, and hospitalization for AECOPD within 1 year before surgical resection were inconsistent between the two groups.

### 3.2. Prognostic Factors for All-Cause Mortality and Colon Cancer Death

Table 1 presents that after PSM, not only all-cause death but also colon cancer death was significantly higher in Group 2 than in Group 1 (*p* < 0.001). A multivariate Cox regression for survival analyses indicated that COPD, hospitalization for AECOPD before surgical resection, age of >60 years, high CCI score, cigarette smoking, high grade of differentiation, perineural invasion, lymphovascular invasion, and advanced AJCC pathologic stages were associated with poor overall survival (OS) (Table 2). No significant differences were observed in explanatory variables, including sex, diabetes, hyperlipidemia, hypertension, ESRD, coronary artery disease, urbanization level, obesity, and chemotherapy (Table 2). In the multivariate Cox regression analysis, the adjusted hazard ratio (aHR) (95% confidence interval (CI)) for all-cause mortality for COPD patients compared with non-COPD patients was 1.17 (1.03, 1.29; *p* = 0.025). Compared with patients with COPD without AECOPD-associated hospitalization within 1 year before surgical resection, the aHR (95% CI) for all-cause mortality for those who were hospitalized for AECOPD once was 1.08 (1.03, 1.51; *p* = 0.031), whereas the aHR (95% CI) of all-cause mortality for those who were hospitalized for AECOPD more than once was 1.55 (1.15, 2.09; *p* = 0.004).

### 3.3. Kaplan–Meier OS among Non-COPD, COPD, and Hospitalization for AECOPD

**Figure 1:**
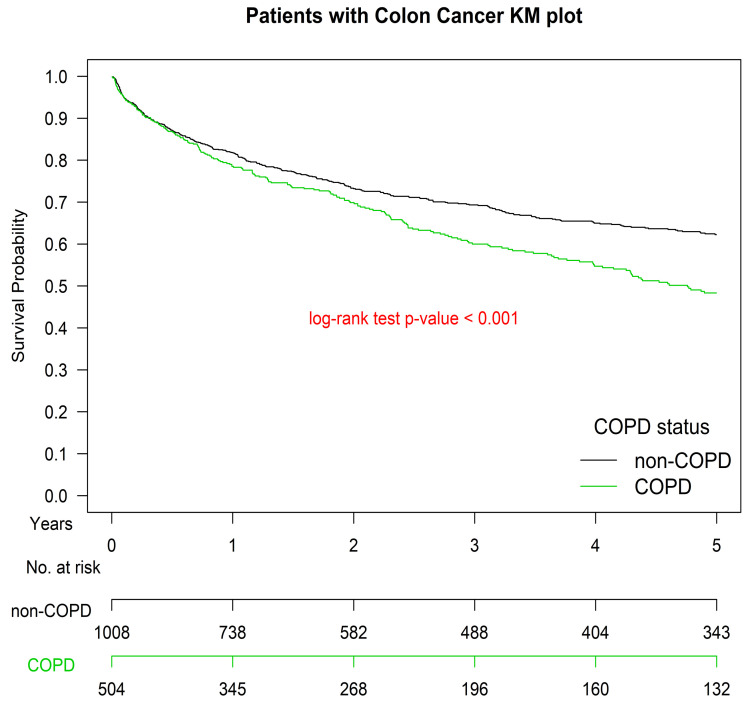
Kaplan–Meier overall survival curves of patients with colon adenocarcinoma with and without chronic obstructive pulmonary disease before surgical resection. COPD, chronic obstruction pulmonary disease; KM, Kaplan–Meier.

**Figure 2:**
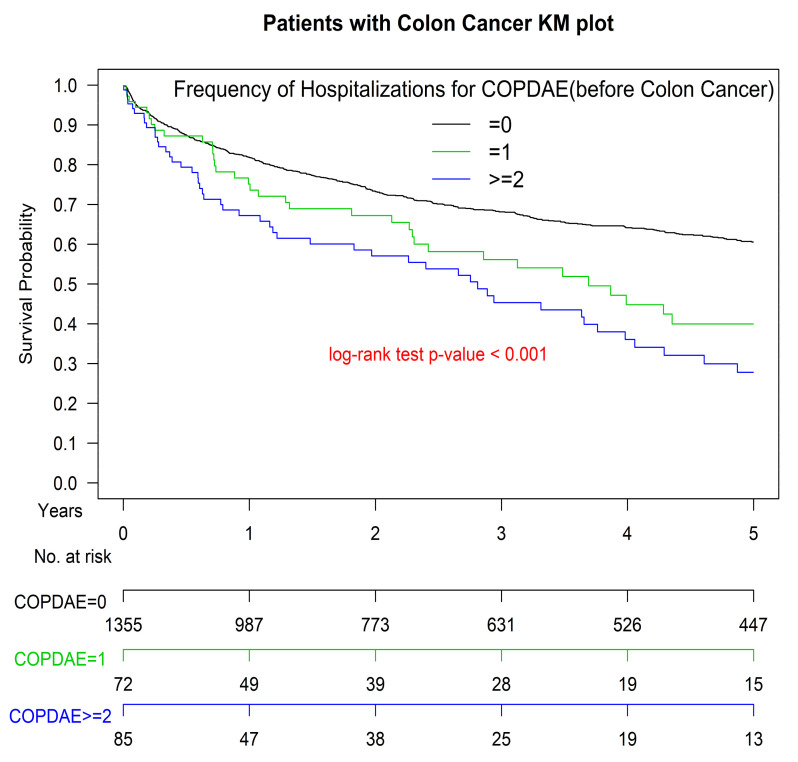
Kaplan–Meier overall survival curves of patients with colon adenocarcinoma and their AECOPD hospitalization frequency within 1 year before surgical resection. COPD, chronic obstruction pulmonary disease; AECOPD, acute exacerbation of COPD; KM, Kaplan–Meier.

The OS curve of patients with COPD was inferior to that of those without COPD (*p* < 0.001) (Figure 1). The Kaplan–Meier OS curve for patients with COPD hospitalized for AECOPD once within 1 year before surgical resection was inferior to that of those not hospitalized for AECOPD; the curve for those with more frequent AECOPD hospitalizations (≥2) was the worst of all study groups (*p* < 0.001) (Figure 2).

## 4. Discussion

This study demonstrated a significant relationship between COPD and colon cancer mortality (Table 2, Figure 1 and Figure 2). The mortality risk increases in patients hospitalized for AECOPD. People hospitalized more than 1 a year before surgical resection have the highest risk. Before this study, no head-to-head PSM study had been conducted to estimate the OS of patients with colon cancer with different COPD histories and severity after they received surgical resection and standard treatments according to the National Comprehensive Cancer Network (NCCN) Clinical Practice Guidelines in Oncology (NCCN Guidelines [23]).

Our study indicated that COPD affects the long-term survival of patients diagnosed as having stage I–III colon cancer and receiving standard treatments. This is consistent with the findings of previous studies, which have investigated the association between COPD and colorectal cancer mortality [24,25]. Furthermore, we showed that hospitalization for AECOPD within 1 year before surgical resection was an independent prognostic factor for survival in patients receiving standard treatments for stage I–III colon adenocarcinoma; patients with more frequent hospitalizations for AECOPD (≥2 within 1 year before surgical resection) before colon adenocarcinoma treatments had poorer survival outcomes than did those with less frequent AECOPD hospitalizations before colon adenocarcinoma treatments. In addition, our study results are compatible with those of previous studies, which have identified age, comorbidity, cigarette smoking status, histological differentiation, perineural invasion, lymphovascular invasion, and AJCC pathologic stages as independent predictors of OS [26,27,28,29,30]. The present study is the first to identify hospitalization frequency for AECOPD before colon adenocarcinoma treatments as a severity-dependent independent prognostic factor for the OS of patients with stage I–III colon cancer receiving surgical resection and standard treatments. Although the mechanisms behind our findings require further research, the significance of our findings should be considered in future medical practice and clinical trials concerning colon cancer and COPD. To increase the OS of patients with colon adenocarcinoma, progression prevention from COPD to AECOPD is crucial according to our findings. Moreover, COPD is an independent poor prognostic factor of the OS of patients undergoing colon adenocarcinoma treatments; this finding is compatible with those of previous studies [24,25]. Therefore, during the treatment of patients with colon adenocarcinoma and COPD, prognostic factors for poor OS should be considered as survival indicators in future clinical trials.

One possible mechanism by which COPD lowers the survival rate of patients with colon adenocarcinoma is spillover inflammation, namely a spillover of the airway inflammation into systemic circulation, resulting in increased concentrations of various inflammatory markers [31]. Moreover, the activation of inflammation cytokines such as NF-κB in malignant cells increases the expression of genes whose products promote cancer cell survival and proliferation [32,33]. Therefore, colon cancer death rates are significantly higher in Group 2 based on the aforementioned possible reasons (Table 1). Studies have demonstrated that inflammation contributes to tumorigenesis, and therapies that regulate inflammation inhibit tumor growth and improve survival [24,34]. Patients with COPD have higher serum concentrations of inflammatory markers, such as C-reactive protein (CRP) [35,36,37], fibrinogen [38], interleukin (IL)-6 [35,36], and IL-8 [36]. In patients with colorectal cancer, increased serum CRP concentrations have been linked to poorer survival [39,40]. In one study, patients with hyperfibrinogen (≥2.61 g/L) had lower 5-year overall and disease-free survival rates [41]. In another study, high serum levels of IL-6 were associated with the tumor stage, tumor size, tumor metastasis, and survival of patients [42]. Increased serum levels of IL-8 because of polymorphisms in the IL-8/CXCR2 genes were associated with disease progression, disease recurrence, and drug resistance [43,44,45,46]. Furthermore, patients with COPD with frequent exacerbations were reported to have high levels of inflammatory markers, such as CRP [47], fibrinogen [47], IL-6 [48], soluble tumor necrosis factor-α receptors [37,49,50], and osteoprotegrin [37], which have been shown to be associated with the survival of patients with colorectal cancer [39,40,41,42,51,52,53,54]. In summary, the findings of our study are in line with those of previous studies regarding the COPD spillover hypothesis.

Another possible reason for our findings is the association between lung function decline and the survival of patients with colon cancer. A recent retrospective study of 1375 patients revealed that patients with colorectal cancer who had ventilatory insufficiency—namely an increase in the minute ventilation to carbon dioxide excretion ratio measured through cardiopulmonary exercise testing—was associated with a decreased long-term survival rate up to 5 years after surgery [25]. Conditions affecting ventilatory efficiency include heart failure, senescence, pulmonary hypertension, chronic smoking, interstitial lung diseases, and COPD [55,56]. Ventilatory inefficiency may develop before a patient is diagnosed as having COPD and worsen along with a decrease in forced expiratory volume in 1 s (FEV_1_) [55]. The relationship between AECOPD frequency and FEV_1_ decline has been observed in multiple studies [57,58], and the frequency of hospitalization for AECOPD within 1 year before commencement of standard colon cancer treatments is associated with long-term survival in these patients.

A previous study conducted by van de Schans et al. has reported that the overall mortality for colon cancer patients with COPD was higher than that for others without COPD, and the difference is more significant in those aged 65 and above [59]. However, cancer stages and consistent cancer treatments (major confounding factors of mortality) were not specified in the study [59]. Moreover, information about COPD severity (defined as the frequency of AECOPD associated with hospitalization within 1 year before surgical resection in our study) was not included either [59]. Our study investigated whether COPD and COPD severity affect the survival of patients with colon cancer receiving standard treatments. COPD severity before colon cancer treatments may be a crucial prognostic factor for survival. Therefore, determining COPD severity and preventing COPD from progressing to AECOPD before colon cancer treatments may be crucial to improving survival after colon cancer treatments.

### 4.1. Strength

The main strength of our study is its novelty as no previous studies have demonstrated that not only a diagnosis of COPD but also COPD severity affects the survival of patients with colon cancer receiving standard treatments. This is the first and largest head-to-head PSM study to estimate the effects of pre-existing COPD and COPD severity on the survival outcomes of patients with colon adenocarcinoma who underwent surgical resection and standard treatments according to the NCCN guidelines. Another strength is that this study addresses the importance of modifying COPD progression from an oncological perspective; a relevant prospective study may never be permitted because of ethical concerns. Moreover, all prognostic factors identified in the literature for the OS of patients with colon cancer were evaluated in our study if the data were available in the TCRD. Before comparing all-cause mortality, we used PSM to minimize confounding effects. The covariates between the COPD and non-COPD groups were homogenous for patients with stage I–III colon cancer receiving standard treatments, and no selection bias was noted between the two groups (Table 1).

### 4.2. Limitations

The results of our study should be interpreted with caution because of the following limitations. First, selection bias and residual or unmeasured confounding (e.g., elective or urgent operation, adjuvant regimens of chemotherapy or radiotherapy, laparoscopic or open surgery, left- or right-sided colon cancer, and different hospital volume) are likely, as in all retrospective studies. Second, data that may be of importance in terms of mortality risk, such as dietary habits, socioeconomic status, or body mass index, are not collected in the TCRD. Third, ethnic differences in both COPD susceptibility and colon adenocarcinoma survival have been reported in relevant studies [60,61]. As such, our results and conclusions may not be extrapolated to non-Asian populations because only Asian patients with colon adenocarcinoma were enrolled in this study. Further large-scale studies comprising various ethnic groups may provide more information on whether different strategies are necessary to improve the survival of patients with colon cancer in each group. Fourth, not all data are recorded accurately in the TCRD. Although data recorded cannot be completely accurate, the TCR long-form data have a high degree of accuracy, probably because these databases periodically undergo field data audits based on medical chart reviews and patient interviews [62]. Despite these limitations, a major strength of this study is the use of a nationwide population-based registry with detailed baseline and treatment information. Lifelong follow-up was possible through the linkage of the registry with the national Cause of Death database. Considering the magnitude and statistical significance of the observed effects in the current study, the limitations are unlikely to affect our conclusions.

## 5. Conclusions

Our data demonstrated that the OS of patients with colon cancer undergoing surgical resection and receiving standard treatments was worse in those with COPD than in those without COPD. When hospitalization frequency for AECOPD within 1 year before surgical resection was considered, an independent frequency-dependent risk was observed in predicting the OS of patients with colon cancer receiving standard treatments. Our study may accentuate the importance of COPD management, particularly the identification of frequent exacerbations and AECOPD prevention before standard treatments in patients with colon adenocarcinoma.

## Figures and Tables

**Table 1:** Characteristics of patients with colon adenocarcinoma with or without chronic obstructive pulmonary disease before surgical resection after propensity score matching.

	Non-COPD Patients		COPD Patients		*p* Value
	N = 1008	, %	N = 504	, %	
Age (mean ± SD)	(72.66 ± 10.14)		(72.26 ± 10.29)		0.854
Age (years)					0.926
Age ≤ 50	212	21.03%	107	21.23%	
50 < age ≤ 60	348	34.52%	174	34.52%	
60 < age ≤ 70	359	35.62%	179	35.52%	
Age > 70	89	8.83%	44	8.73%	
Sex					0.924
Female	358	35.52%	177	35.12%	
Male	650	64.48%	327	64.88%	
Diabetes					0.985
No	658	65.28%	328	65.08%	
Yes	350	34.72%	176	34.92%	
Hyperlipidemia					0.752
No	604	59.92%	307	60.91%	
Yes	404	40.08%	197	39.09%	
Hypertension					0.951
No	605	60.02%	303	60.12%	
Yes	403	39.98%	201	39.88%	
ESRD					0.288
No	977	96.92%	494	98.02%	
Yes	31	3.08%	10	1.98%	
Cigarette Smoking					0.940
No	302	29.96%	148	29.37%	
Yes	706	70.04%	356	70.63%	
Coronary heart disease					0.234
No	863	85.62%	419	83.13%	
Yes	145	14.38%	85	16.87%	
Obesity					0.842
No	898	89.09%	448	88.89%	
Yes	110	10.91%	56	11.11%	
CCI score					0.779
0	618	61.31%	305	60.52%	
≥1	390	38.69%	199	39.48%	
Differentiation					0.135
I	92	9.13%	59	11.71%	
II	620	61.51%	302	59.92%	
III	296	29.37%	143	28.37%	
Urbanization					0.858
Rural	296	29.37%	151	29.96%	
Urban	712	70.63%	353	70.04%	
Perineural invasion					0.122
No	379	37.60%	211	41.87%	
Yes	629	62.40%	293	58.13%	
Chemotherapy					0.783
No	766	75.99%	379	75.20%	
Yes	242	24.01%	125	24.80%	
Lymphovascular invasion					0.941
No	555	55.06%	277	54.96%	
Yes	453	44.94%	227	45.04%	
AJCC pathologic stages					1.000
I	280	27.78	140	27.78	
IIA	112	11.11	56	11.11	
IIB–IIC	224	22.22	112	22.22	
IIIA	134	13.29	67	13.29	
IIIB–IIIC	258	25.60	129	25.60	
All-cause death					**0.039**
No	582	57.74%	262	51.98%	
Yes	426	42.26%	242	48.02%	
Colon cancer death					**<0.001**
No	635	63.00%	272	53.97	
Yes	373	37.00%	232	46.03%	
Follow up (death) Years, median (IQR, Q1, Q3)	2.80 (0.86, 6.84)		2.13 (0.73, 5.35)		**0.002**
Follow up (death) Years, (mean ± SD)	(4.33 ± 4.29)		(3.36 ± 3.34)		**<0.001**
Hospitalizations frequency for AECOPD (before colon cancer)					**<0.001**
0	1008	100.00%	347	68.85%	
1	0	0.00%	72	14.29%	
≥2	0	0.00%	85	16.87%	

IQR, interquartile range; SD, standard deviation; AJCC, American Joint Committee on Cancer; CCI, Charlson comorbidity index; COPD, chronic obstruction pulmonary disease; AECOPD, acute exacerbation of COPD; ESRD, end stage renal disease.

**Table 2:** Cox proportional hazards models for all-cause mortality of patients with colon adenocarcinoma with or without chronic obstructive pulmonary disease before surgical resection after propensity-score matching.

Variables	Crude HR (95% CI)		Adjusted HR^*^ (95% CI)		*p* Value
COPD status (ref. non-COPD)					
COPD	1.35	(1.15, 1.58)	1.17	(1.03, 1.29)	**0.025**
Hospitalization frequency for AECOPD before surgical resection (ref. = 0)					
1	1.60	(1.15, 2.21)	1.08	(1.03, 1.51)	**0.031**
≥2	2.36	(1.81, 3.08)	1.55	(1.15, 2.09)	**0.004**
Sex (ref. female)					
Male	0.90	(0.77, 1.06)	0.89	(0.75, 1.05)	0.152
Age (ref. ≤ 50 years)					
50 years < age ≤ 60 years	1.21	(0.93, 1.56)	1.05	(0.80, 1.37)	0.714
60 years < age ≤ 70 years	2.38	(1.88, 3.02)	2.07	(1.61, 2.67)	**<0.001**
Age > 70 years	3.99	(2.98, 5.34)	3.29	(2.41, 4.50)	**<0.001**
CCI score (ref. = 0)					
≥1	1.58	(1.36, 1.84)	1.46	(1.23, 1.72)	**<0.001**
Diabetes (ref.: No)					
Yes	1.30	(0.91, 1.53)	1.14	(0.84, 1.48)	0.315
Hyperlipidemia (ref.: No)					
Yes	0.96	(0.81, 1.13)	0.91	(0.76, 1.09)	0.317
Hypertension (ref.: No)					
Yes	0.96	(0.81, 1.14)	0.94	(0.79, 1.13)	0.537
ESRD (ref.: No)					
Yes	1.42	(1.05, 1.92)	0.93	(0.67, 1.27)	0.642
Coronary heart disease (ref.: No)					
Yes	1.47	(1.2, 1.8)	1.11	(0.84, 1.45)	0.463
Cigarette smoking (ref.: No)					
Yes	1.68	(1.36, 2.06)	1.27	(0.96, 1.68)	**0.007**
Differentiation (ref. grade I)					
II	2.14	(1.33, 3.43)	1.50	(1.11, 1.49)	**0.014**
III	2.79	(1.76, 4.42)	1.92	(1.18, 2.31)	**0.008**
Urbanization (ref. urban)					
Rural	0.94	(0.80, 1.11)	0.92	(0.76, 1.10)	0.348
Obesity (ref.: No)					
Yes	0.93	(0.45, 1.42)	0.95	(0.47, 1.45)	0.752
Chemotherapy (ref.: No)					
Yes	1.17	(0.84, 1.64)	1.15	(0.92, 1.25)	0.479
Perineural invasion (ref.: No)					
Yes	1.72	(1.25, 2.36)	1.48	(1.06, 2.07)	**0.020**
Lymphovascular invasion (ref.: No)					
Yes	1.30	(1.11, 1.53)	1.24	(1.04, 1.48)	**0.015**
AJCC pathologic stage (ref.: stage I)					
IIA	1.14	(1.03, 1.43)	1.04	(0.93, 1.36)	0.092
IIB–IIC	1.19	(1.06, 1.42)	1.15	(1.10, 1.36)	**0.007**
IIIA	2.14	(1.33, 2.73)	1.54	(1.31, 2.56)	**0.002**
IIIB–IIIC	2.72	(1.25, 2.96)	2.10	(1.38, 2.80)	**0.006**

HR, hazard ratio; CI, confidence interval; AJCC, American Joint Committee on Cancer; CCI, Charlson comorbidity index; COPD, chronic obstruction pulmonary disease; AECOPD, acute exacerbation of COPD; ref., reference group; ESRD, end-stage renal disease * All covariates mentioned in Table 2 were adjusted.

## Data Availability

Informed consent was waived because the data sets are covered under the Personal Information Protection Act. We used data from the National Health Insurance Research Database (NHIRD) and Taiwan Cancer Registry database (TCRD). The authors confirm that, for approved reasons, some access restrictions apply to the data underlying the findings. The data utilized in this study cannot be made available in the manuscript, the supplemental files, or in a public repository due to the “Personal Information Protection Act” executed by Taiwan’s government, starting from 2012. Requests for data can be sent as a formal proposal to obtain approval from the ethics review committee of the appropriate governmental department in Taiwan. Specifically, links regarding contact info for which data requests may be sent to the following web address (accessed date: 29 December 2020): http://nhird.nhri.org.tw/en/Data_Subsets.html#S3 and http://nhis.nhri.org.tw/point.html. Szu-Yuan Wu, had full access to all the data in the study and takes responsibility for the integrity of the data and the accuracy of the data analysis.

## Abbreviations

COPD: chronic obstructive pulmonary disease; AECOPD, acute exacerbation of COPD; aHR, adjusted hazard ratio; CI, confidence interval; AJCC, American Joint Committee on Cancer; TCRD, Taiwan Cancer Registry Database; PSM, propensity score matching; OS, overall survival; CRP, C-reactive protein; IL, interleukin; CCI, Charlson comorbidity index; ESRD, end-stage renal disease; NCCN, National Comprehensive Cancer Network; FEV1, forced expiratory volume in 1 s.
